# Supplementary material for: Heparin-based hydrogel scaffolding alters the transcriptomic profile and increases the chemoresistance of MDA-MB-231 triple-negative breast cancer cells
Source: Biomater Sci. 2020 Feb 13;8(10):2786–96. doi: 10.1039/c9bm01481k (PMC7497406; doi:10.1039/c9bm01481k)
Supplement: Supplementary file 2 [file BM-008-C9BM01481K-s002.zip › Supplementary File 4/HP-BhydrogelvControl/Pathways/my_analysis.Gsea.1545201079907/HALLMARK_MITOTIC_SPINDLE.html]

Details for gene set HALLMARK\_MITOTIC\_SPINDLE[GSEA]

|  || Dataset | expr.class.cls#HYD\_versus\_CONTROL.class.cls#HYD\_versus\_CONTROL\_repos |
| Phenotype | class.cls#HYD\_versus\_CONTROL\_repos |
| Upregulated in class | HYD |
| GeneSet | HALLMARK\_MITOTIC\_SPINDLE |
| Enrichment Score (ES) | 0.2364175 |
| Normalized Enrichment Score (NES) | 0.9702316 |
| Nominal p-value | 0.5253623 |
| FDR q-value | 0.87537783 |
| FWER p-Value | 1.0 |
Table: GSEA Results Summary

  

Fig 1: Enrichment plot: HALLMARK\_MITOTIC\_SPINDLE      
 Profile of the Running ES Score & Positions of GeneSet Members on the Rank Ordered List

  

| PROBE | DESCRIPTION (from dataset) | GENE SYMBOL | GENE\_TITLE | RANK IN GENE LIST | RANK METRIC SCORE | RUNNING ES | CORE ENRICHMENT || 1 | CEP250 | na |  |  | 281 | 2.703 | 0.0038 | Yes |
| 2 | TAOK2 | na |  |  | 355 | 2.559 | 0.0175 | Yes |
| 3 | MYO9B | na |  |  | 445 | 2.419 | 0.0295 | Yes |
| 4 | CLIP2 | na |  |  | 498 | 2.352 | 0.0429 | Yes |
| 5 | NUMA1 | na |  |  | 566 | 2.257 | 0.0549 | Yes |
| 6 | TUBGCP6 | na |  |  | 669 | 2.145 | 0.0642 | Yes |
| 7 | BCR | na |  |  | 702 | 2.112 | 0.0770 | Yes |
| 8 | ATG4B | na |  |  | 735 | 2.089 | 0.0897 | Yes |
| 9 | ABL1 | na |  |  | 912 | 1.940 | 0.0938 | Yes |
| 10 | KLC1 | na |  |  | 972 | 1.892 | 0.1037 | Yes |
| 11 | ARHGAP27 | na |  |  | 1011 | 1.868 | 0.1145 | Yes |
| 12 | CNTROB | na |  |  | 1031 | 1.853 | 0.1262 | Yes |
| 13 | HDAC6 | na |  |  | 1073 | 1.826 | 0.1366 | Yes |
| 14 | CTTN | na |  |  | 1199 | 1.742 | 0.1420 | Yes |
| 15 | ARHGEF11 | na |  |  | 1432 | 1.614 | 0.1409 | Yes |
| 16 | ARAP3 | na |  |  | 1454 | 1.601 | 0.1508 | Yes |
| 17 | RHOT2 | na |  |  | 1503 | 1.574 | 0.1591 | Yes |
| 18 | BCAR1 | na |  |  | 1577 | 1.541 | 0.1658 | Yes |
| 19 | SUN2 | na |  |  | 1585 | 1.539 | 0.1760 | Yes |
| 20 | ARHGAP4 | na |  |  | 1874 | 1.416 | 0.1706 | Yes |
| 21 | PCNT | na |  |  | 2294 | 1.239 | 0.1571 | Yes |
| 22 | KATNB1 | na |  |  | 2499 | 1.172 | 0.1544 | Yes |
| 23 | SPTAN1 | na |  |  | 2525 | 1.164 | 0.1611 | Yes |
| 24 | MAP3K11 | na |  |  | 2629 | 1.135 | 0.1635 | Yes |
| 25 | CSNK1D | na |  |  | 2706 | 1.113 | 0.1671 | Yes |
| 26 | CYTH2 | na |  |  | 2745 | 1.105 | 0.1727 | Yes |
| 27 | SEPT9 | na |  |  | 2779 | 1.092 | 0.1785 | Yes |
| 28 | SYNPO | na |  |  | 2788 | 1.089 | 0.1855 | Yes |
| 29 | DLG1 | na |  |  | 2806 | 1.083 | 0.1921 | Yes |
| 30 | BIN1 | na |  |  | 2965 | 1.045 | 0.1909 | Yes |
| 31 | TUBGCP2 | na |  |  | 3007 | 1.035 | 0.1959 | Yes |
| 32 | CDK5RAP2 | na |  |  | 3110 | 1.007 | 0.1974 | Yes |
| 33 | PKD2 | na |  |  | 3226 | 0.982 | 0.1981 | Yes |
| 34 | PXN | na |  |  | 3272 | 0.969 | 0.2024 | Yes |
| 35 | KPTN | na |  |  | 3423 | 0.933 | 0.2009 | Yes |
| 36 | TUBGCP5 | na |  |  | 3449 | 0.928 | 0.2060 | Yes |
| 37 | ALS2 | na |  |  | 3479 | 0.923 | 0.2108 | Yes |
| 38 | PLEKHG2 | na |  |  | 3546 | 0.908 | 0.2136 | Yes |
| 39 | RASAL2 | na |  |  | 3579 | 0.898 | 0.2181 | Yes |
| 40 | TBCD | na |  |  | 3819 | 0.847 | 0.2113 | Yes |
| 41 | CEP72 | na |  |  | 3904 | 0.832 | 0.2126 | Yes |
| 42 | SHROOM1 | na |  |  | 4058 | 0.799 | 0.2100 | Yes |
| 43 | MAP1S | na |  |  | 4172 | 0.775 | 0.2094 | Yes |
| 44 | ARHGAP10 | na |  |  | 4204 | 0.771 | 0.2131 | Yes |
| 45 | SHROOM2 | na |  |  | 4267 | 0.758 | 0.2150 | Yes |
| 46 | TUBD1 | na |  |  | 4356 | 0.741 | 0.2155 | Yes |
| 47 | ALMS1 | na |  |  | 4476 | 0.720 | 0.2142 | Yes |
| 48 | MARK4 | na |  |  | 4538 | 0.704 | 0.2158 | Yes |
| 49 | CENPJ | na |  |  | 4549 | 0.702 | 0.2201 | Yes |
| 50 | INCENP | na |  |  | 4652 | 0.685 | 0.2194 | Yes |
| 51 | SMC1A | na |  |  | 4737 | 0.670 | 0.2196 | Yes |
| 52 | PIF1 | na |  |  | 4748 | 0.668 | 0.2237 | Yes |
| 53 | MARCKS | na |  |  | 4831 | 0.652 | 0.2238 | Yes |
| 54 | GEMIN4 | na |  |  | 4888 | 0.644 | 0.2253 | Yes |
| 55 | RANBP9 | na |  |  | 4894 | 0.643 | 0.2295 | Yes |
| 56 | DOCK2 | na |  |  | 4960 | 0.634 | 0.2304 | Yes |
| 57 | NCK2 | na |  |  | 5037 | 0.621 | 0.2307 | Yes |
| 58 | ESPL1 | na |  |  | 5108 | 0.612 | 0.2312 | Yes |
| 59 | KIF22 | na |  |  | 5147 | 0.606 | 0.2334 | Yes |
| 60 | LLGL1 | na |  |  | 5169 | 0.604 | 0.2364 | Yes |
| 61 | FARP1 | na |  |  | 5271 | 0.589 | 0.2352 | No |
| 62 | CDC42BPA | na |  |  | 5539 | 0.547 | 0.2249 | No |
| 63 | TUBGCP3 | na |  |  | 5601 | 0.538 | 0.2254 | No |
| 64 | CEP192 | na |  |  | 5738 | 0.521 | 0.2218 | No |
| 65 | FGD4 | na |  |  | 6090 | 0.472 | 0.2066 | No |
| 66 | CLASP1 | na |  |  | 6128 | 0.467 | 0.2078 | No |
| 67 | SAC3D1 | na |  |  | 6253 | 0.451 | 0.2044 | No |
| 68 | EPB41L2 | na |  |  | 6259 | 0.449 | 0.2072 | No |
| 69 | ARHGDIA | na |  |  | 6491 | 0.417 | 0.1979 | No |
| 70 | RHOF | na |  |  | 6593 | 0.404 | 0.1954 | No |
| 71 | MYH10 | na |  |  | 6659 | 0.398 | 0.1947 | No |
| 72 | EZR | na |  |  | 6694 | 0.394 | 0.1956 | No |
| 73 | ABR | na |  |  | 6854 | 0.377 | 0.1899 | No |
| 74 | NF1 | na |  |  | 6979 | 0.364 | 0.1858 | No |
| 75 | AKAP13 | na |  |  | 7039 | 0.355 | 0.1852 | No |
| 76 | ARHGEF2 | na |  |  | 7083 | 0.350 | 0.1853 | No |
| 77 | CNTRL | na |  |  | 7211 | 0.338 | 0.1809 | No |
| 78 | SSH2 | na |  |  | 7337 | 0.321 | 0.1766 | No |
| 79 | KIF3C | na |  |  | 7585 | 0.296 | 0.1656 | No |
| 80 | KNTC1 | na |  |  | 7885 | 0.263 | 0.1517 | No |
| 81 | CDC42EP1 | na |  |  | 8086 | 0.248 | 0.1429 | No |
| 82 | CLIP1 | na |  |  | 8103 | 0.246 | 0.1437 | No |
| 83 | ARL8A | na |  |  | 8310 | 0.218 | 0.1344 | No |
| 84 | NOTCH2 | na |  |  | 8394 | 0.208 | 0.1315 | No |
| 85 | DOCK4 | na |  |  | 9066 | 0.145 | 0.0972 | No |
| 86 | ROCK1 | na |  |  | 9075 | 0.144 | 0.0977 | No |
| 87 | TSC1 | na |  |  | 9081 | 0.144 | 0.0984 | No |
| 88 | ITSN1 | na |  |  | 9112 | 0.139 | 0.0978 | No |
| 89 | LMNB1 | na |  |  | 9190 | 0.127 | 0.0946 | No |
| 90 | PAFAH1B1 | na |  |  | 9194 | 0.126 | 0.0954 | No |
| 91 | TUBA4A | na |  |  | 10073 | 0.033 | 0.0494 | No |
| 92 | ARHGEF12 | na |  |  | 10414 | 0.006 | 0.0315 | No |
| 93 | FSCN1 | na |  |  | 10428 | 0.004 | 0.0309 | No |
| 94 | CDC42EP2 | na |  |  | 10655 | -0.024 | 0.0192 | No |
| 95 | KIF3B | na |  |  | 10682 | -0.027 | 0.0180 | No |
| 96 | PLK1 | na |  |  | 10895 | -0.053 | 0.0072 | No |
| 97 | DYNLL2 | na |  |  | 10898 | -0.054 | 0.0075 | No |
| 98 | ARHGEF7 | na |  |  | 10994 | -0.064 | 0.0029 | No |
| 99 | FGD6 | na |  |  | 11072 | -0.074 | -0.0006 | No |
| 100 | SMC4 | na |  |  | 11306 | -0.090 | -0.0123 | No |
| 101 | KIF4A | na |  |  | 11399 | -0.101 | -0.0164 | No |
| 102 | NIN | na |  |  | 11437 | -0.106 | -0.0176 | No |
| 103 | EPB41 | na |  |  | 11440 | -0.106 | -0.0170 | No |
| 104 | CDC42EP4 | na |  |  | 11452 | -0.108 | -0.0169 | No |
| 105 | ARHGEF3 | na |  |  | 11588 | -0.124 | -0.0231 | No |
| 106 | CD2AP | na |  |  | 11684 | -0.136 | -0.0272 | No |
| 107 | KIF2C | na |  |  | 11924 | -0.166 | -0.0386 | No |
| 108 | KIF15 | na |  |  | 12046 | -0.179 | -0.0437 | No |
| 109 | BUB1 | na |  |  | 12059 | -0.180 | -0.0431 | No |
| 110 | KIF1B | na |  |  | 12121 | -0.189 | -0.0450 | No |
| 111 | KIF23 | na |  |  | 12161 | -0.194 | -0.0457 | No |
| 112 | NEDD9 | na |  |  | 12213 | -0.201 | -0.0471 | No |
| 113 | MYO1E | na |  |  | 12231 | -0.204 | -0.0465 | No |
| 114 | TPX2 | na |  |  | 12233 | -0.204 | -0.0452 | No |
| 115 | ARFIP2 | na |  |  | 12282 | -0.210 | -0.0463 | No |
| 116 | SORBS2 | na |  |  | 12311 | -0.215 | -0.0463 | No |
| 117 | MID1IP1 | na |  |  | 12502 | -0.224 | -0.0547 | No |
| 118 | VCL | na |  |  | 12619 | -0.236 | -0.0592 | No |
| 119 | WASF1 | na |  |  | 12753 | -0.253 | -0.0645 | No |
| 120 | NUSAP1 | na |  |  | 12808 | -0.258 | -0.0655 | No |
| 121 | WASF2 | na |  |  | 12841 | -0.263 | -0.0654 | No |
| 122 | CENPF | na |  |  | 12914 | -0.273 | -0.0673 | No |
| 123 | ANLN | na |  |  | 13065 | -0.293 | -0.0732 | No |
| 124 | KIF11 | na |  |  | 13273 | -0.320 | -0.0819 | No |
| 125 | ARFGEF1 | na |  |  | 13379 | -0.334 | -0.0851 | No |
| 126 | PALLD | na |  |  | 13461 | -0.347 | -0.0870 | No |
| 127 | KIF5B | na |  |  | 13478 | -0.350 | -0.0854 | No |
| 128 | RAPGEF6 | na |  |  | 13529 | -0.357 | -0.0856 | No |
| 129 | RACGAP1 | na |  |  | 13570 | -0.365 | -0.0852 | No |
| 130 | RAPGEF5 | na |  |  | 13662 | -0.379 | -0.0874 | No |
| 131 | DLGAP5 | na |  |  | 13683 | -0.383 | -0.0858 | No |
| 132 | WASL | na |  |  | 13860 | -0.412 | -0.0922 | No |
| 133 | KIF20B | na |  |  | 14040 | -0.422 | -0.0987 | No |
| 134 | RASA1 | na |  |  | 14048 | -0.423 | -0.0962 | No |
| 135 | RABGAP1 | na |  |  | 14145 | -0.434 | -0.0983 | No |
| 136 | CKAP5 | na |  |  | 14303 | -0.464 | -0.1033 | No |
| 137 | BRCA2 | na |  |  | 14335 | -0.469 | -0.1018 | No |
| 138 | ABI1 | na |  |  | 14438 | -0.481 | -0.1038 | No |
| 139 | BCL2L11 | na |  |  | 14467 | -0.486 | -0.1020 | No |
| 140 | RALBP1 | na |  |  | 14489 | -0.488 | -0.0997 | No |
| 141 | AURKA | na |  |  | 14577 | -0.502 | -0.1008 | No |
| 142 | BIRC5 | na |  |  | 14629 | -0.512 | -0.1000 | No |
| 143 | APC | na |  |  | 14635 | -0.512 | -0.0967 | No |
| 144 | ARF6 | na |  |  | 14738 | -0.529 | -0.0985 | No |
| 145 | CDC42 | na |  |  | 14796 | -0.537 | -0.0978 | No |
| 146 | TIAM1 | na |  |  | 14991 | -0.561 | -0.1041 | No |
| 147 | CCDC88A | na |  |  | 15074 | -0.574 | -0.1045 | No |
| 148 | MAPRE1 | na |  |  | 15092 | -0.578 | -0.1014 | No |
| 149 | PPP4R2 | na |  |  | 15095 | -0.578 | -0.0976 | No |
| 150 | PCM1 | na |  |  | 15180 | -0.593 | -0.0979 | No |
| 151 | ECT2 | na |  |  | 15181 | -0.593 | -0.0938 | No |
| 152 | LRPPRC | na |  |  | 15236 | -0.603 | -0.0925 | No |
| 153 | HOOK3 | na |  |  | 15290 | -0.615 | -0.0911 | No |
| 154 | ARHGAP29 | na |  |  | 15454 | -0.642 | -0.0953 | No |
| 155 | GSN | na |  |  | 15576 | -0.665 | -0.0971 | No |
| 156 | RAB3GAP1 | na |  |  | 15585 | -0.666 | -0.0929 | No |
| 157 | RICTOR | na |  |  | 15603 | -0.668 | -0.0892 | No |
| 158 | CENPE | na |  |  | 15857 | -0.715 | -0.0976 | No |
| 159 | NDC80 | na |  |  | 15904 | -0.723 | -0.0951 | No |
| 160 | TOP2A | na |  |  | 15987 | -0.741 | -0.0943 | No |
| 161 | TLK1 | na |  |  | 16010 | -0.748 | -0.0903 | No |
| 162 | PRC1 | na |  |  | 16085 | -0.766 | -0.0889 | No |
| 163 | MID1 | na |  |  | 16162 | -0.780 | -0.0876 | No |
| 164 | STAU1 | na |  |  | 16629 | -0.881 | -0.1060 | No |
| 165 | SMC3 | na |  |  | 16746 | -0.913 | -0.1059 | No |
| 166 | SASS6 | na |  |  | 16862 | -0.945 | -0.1054 | No |
| 167 | CDK1 | na |  |  | 16949 | -0.970 | -0.1033 | No |
| 168 | CAPZB | na |  |  | 17043 | -0.998 | -0.1013 | No |
| 169 | CCNB2 | na |  |  | 17048 | -0.999 | -0.0947 | No |
| 170 | STK38L | na |  |  | 17190 | -1.047 | -0.0949 | No |
| 171 | OPHN1 | na |  |  | 17263 | -1.072 | -0.0913 | No |
| 172 | RASA2 | na |  |  | 17300 | -1.084 | -0.0858 | No |
| 173 | PDLIM5 | na |  |  | 17595 | -1.194 | -0.0930 | No |
| 174 | LATS1 | na |  |  | 17635 | -1.215 | -0.0867 | No |
| 175 | PCGF5 | na |  |  | 17641 | -1.217 | -0.0786 | No |
| 176 | FBXO5 | na |  |  | 17725 | -1.253 | -0.0744 | No |
| 177 | SOS1 | na |  |  | 17850 | -1.309 | -0.0719 | No |
| 178 | RFC1 | na |  |  | 17908 | -1.336 | -0.0658 | No |
| 179 | NEK2 | na |  |  | 17957 | -1.352 | -0.0590 | No |
| 180 | NET1 | na |  |  | 18043 | -1.410 | -0.0538 | No |
| 181 | CEP57 | na |  |  | 18047 | -1.414 | -0.0442 | No |
| 182 | KIFAP3 | na |  |  | 18109 | -1.448 | -0.0375 | No |
| 183 | CDC27 | na |  |  | 18397 | -1.639 | -0.0413 | No |
| 184 | YWHAE | na |  |  | 18488 | -1.699 | -0.0344 | No |
| 185 | NCK1 | na |  |  | 18624 | -1.812 | -0.0290 | No |
| 186 | UXT | na |  |  | 18728 | -1.925 | -0.0212 | No |
| 187 | TTK | na |  |  | 18742 | -1.945 | -0.0086 | No |
| 188 | ARHGAP5 | na |  |  | 18764 | -1.967 | 0.0038 | No |
| 189 | KATNA1 | na |  |  | 19102 | -2.756 | 0.0050 | No |
Table: GSEA details [plain text format]

  

Fig 2: HALLMARK\_MITOTIC\_SPINDLE      
 Blue-Pink O' Gram in the Space of the Analyzed GeneSet

  

Fig 3: HALLMARK\_MITOTIC\_SPINDLE: Random ES distribution      
 Gene set null distribution of ES for **HALLMARK\_MITOTIC\_SPINDLE**
